# Downregulation of EVI1 Expression Inhibits Cell Proliferation and Induces Apoptosis in Hilar Cholangiocarcinoma via the PTEN/AKT Signalling Pathway

**DOI:** 10.7150/jca.31903

**Published:** 2020-01-13

**Authors:** Xiao-ming Zhang, Zeng-li Liu, Bo Qiu, Yun-fei XU, Chang Pan, Zong-li Zhang

**Affiliations:** 1 Department of general surgery, Qilu Hospital of Shandong University, No. 107, Wenhua Xi Road, Jinan, 250012, China.; 2 Department of general surgery, Linyi People's Hospital, Linyi, 276000, China.; 3 Department of general surgery, Qilu Hospital of Shandong University (Qingdao), 266035, China.; 4 Department of emergency, Qilu Hospital of Shandong University, No. 107, Wenhua Xi Road, Jinan, 250012, China.

**Keywords:** Hilar cholangiocarcinoma, EVI1, Cell proliferation, PTEN

## Abstract

**Aims**: Hilar cholangiocarcinoma (HCCA) is a tumour with high malignancy, low surgical resection potential, and a poor prognosis. Ecotropic Viral Integration site 1 (EVI1) is a transcriptional regulator that has been proven to be associated with tumourigenesis and progression in many human solid tumours. However, the expression of EVI1 and its role in HCCA progression remain unclear. The aim of this study was to clarify the association between EVI1 expression and clinical outcomes in patients with HCCA.

**Methods**: The expression of EVI1 in HCCA tissue samples and cell lines was examined by quantitative real-time PCR (qRT-PCR), Western blotting, and immunohistochemistry (IHC). Kaplan-Meier analysis was used for survival analysis. A log-rank test was performed for univariate analysis of survival, and a Cox regression model was utilized for multivariate analysis of survival. Cell proliferation was measured by cell counting kit-8 (CCK-8), colony formation, and 5-ethynyl-2'-deoxyuridine (EdU) assays. The cell cycle was evaluated by flow cytometry. Cell apoptosis was detected by flow cytometry and a terminal deoxynucleotidyl transferase (TdT)-mediated dUTP nick-end labelling (TUNEL) assay. In vivo tumour growth was observed for xenografts in nude mice.

**Results**: EVI1 expression was upregulated in HCCA tissue samples and correlated with a poor prognosis. In clinical specimens, the expression of EVI1 correlated with tumour histological grade and tumour size. Knocking down EVI1 expression reduced HCCA cell proliferation, blocked cell cycle progression, and promoted apoptosis in vitro and in vivo. Furthermore, we found that EVI1 could regulate the AKT signalling pathway by regulating PTEN levels in HCCA.

**Conclusion**: Our data revealed that EVI1 played important roles in HCCA tumourigenesis and development. Our findings suggest that EVI1 may be a potentially useful therapeutic target in HCCA.

## Introduction

Cholangiocarcinoma (CCA) originates in the epithelial cells of the bile duct wall, and its incidence has increased significantly in the past several decades^1^. Hilar cholangiocarcinoma (HCCA) is the most common type of CCA^2^, accounting for 58-66% of Chinese CCA cases^3^. Surgical resection is still the only curative treatment approach, but it can be applied only to in patients with early-stage HCCA^4^. Unfortunately, most patients with HCCA are diagnosed at an advanced stage, and tumour resection rates are frustratingly low^5^, ^6^. Although the combination of cisplatin and gemcitabine is the standard first-line chemotherapy**,** the efficacy of this treatment regimen is discouraging^3^, ^7^. Therefore, the identification of new signalling molecules involved in the proliferation and progression of HCCA cells is critical for the development of new drugs to treat this disease.

Ecotropic viral integration site 1 (EVI1), which forms a fusion gene with MDS1 named MECOM, is a nuclear transcription factor that is indispensable for normal development and oncogenesis^8^. EVI1 overexpression and activation have been proven to result in genomic instability and clonal evolution in myelodysplastic syndrome (MDS) and eventually lead to acute myeloid leukaemia (AML)^9^, ^10^. In addition to malignancies of haematopoietic origin, some solid tumours, such as melanoma, prostate cancer, breast cancer, and ovarian cancer, also exhibit EVI1 overexpression, and the involvement of EVI1 in the occurrence and/or progression^11^, ^12^ of these tumours has been shown. A recent study showed that the expression of EVI1 was upregulated in over 50% of intrahepatic cholangiocarcinomas (ICCs), and patients with EVI1-high ICC showed worse overall survival than patients with EVI1-low ICC^13^. However, the relevance of EVI1 and HCCA is still elusive. In the present study, we explored the expression and biological function of EVI1 in HCCA.

Although accumulating studies have shown the involvement of EVI1 in tumour progression, the mechanism by which EVI1 promotes tumourigenesis and progression has not been fully elucidated. A recent study demonstrated that EVI1 downregulated Phosphatase and tensin homologue (PTEN) expression and activated the AKT signalling pathway in leukaemia cells, thereby causing malignant cell proliferation^14^. Therefore, EVI1-mediated tumour progression may be attributed to the downregulation of PTEN expression. PTEN is an important tumour suppressor gene that negatively regulates the PI3K/PKB/Akt signalling pathway. Although a decrease in PTEN expression is observed in most solid tumours, genetic mutations in PTEN are quite rare in most cancer types, except glioblastoma multiforme and endometrial cancer^15^. Therefore, the most likely reason for the loss of PTEN activity or protein expression in HCCA or other types of cancer might be downregulation by transcriptional regulation. In our study, the influence of EVI1 on the expression of PTEN in HCCA was evaluated.

Here, we showed that overexpression of EVI1 was associated with poor survival in patients with HCCA. Downregulation of EVI1 expression inhibited proliferation and promoted apoptosis in HCCA cells. This repression may be caused by downregulating PTEN expression and thus inactivating the AKT pathway. EVI1 may be a key potential new target for the treatment of HCCA.

## Materials and Methods

### Tissue samples

Paired HCCA tumour tissue samples and adjacent normal bile duct tissue samples were obtained from 30 consecutive patients who underwent therapeutic resection of HCCA at Qilu Hospital of Shandong University during 2017-2018. The study obtained informed consent from all participants, who were confirmed to have HCCA by pathology. All the specimens were collected in accordance with procedures authorized by the ethics committee of Qilu Hospital of Shandong University and stored at -80℃ until use.

### Human HCCA tissue microarray

A human HCCA tissue microarray (TMA) containing 114 samples of HCCA tissue was immunohistochemically stained for EVI1 and PTEN and analysed for correlations between EVI1 and clinicopathological features. Clinical characteristics of all patients contained in the TMA were collected and are shown in Table 1.

### Cell lines and culture

QBC939 and RBE cells were purchased from the Cell Bank of the Chinese Academy of Sciences (Shanghai, China). Other cell lines, including FRH0201 and HCCC9810, and human intrahepatic biliary epithelial cells (HIBEpiC) were preserved in our laboratory. QBC939 cells and HIBEpiC were cultured in DMEM (Gibco, USA) supplemented with 10% foetal bovine serum (FBS; Gibco) and 1% penicillin/streptomycin. RBE, FRH0201 and HCCC9810 cells were cultured in RPMI-1640 (Gibco) supplemented with 10% FBS (Gibco) and 1% penicillin/streptomycin. All the cells were maintained at 37°C in a humidified incubator under 5% CO2 conditions.

### Stable cell line construction and siRNA interference

EVI1-specific shRNA and noncoding shRNA lentiviral particles were purchased from GeneChem (Shanghai, China). For construction of HCCA with stably downregulated EVI1 expression, the EVI1-specific shRNA and noncoding shRNA lentiviral particles were transfected into QBC939 cells, and then the cells were selected with 2 μg/mL puromycin for 4 weeks. The cells were transfected with Lipofectamine 2000 (Invitrogen) in Opti-MEM (Gibco) according to the manufacturer's instructions. The EVI1 overexpressing plasmid and a PTEN-specific siRNA were purchased from GenePharma (Shanghai, China). The siRNA sequence targeting PTEN was as follows: 5'-GAAGAUAUAUUCCUCCAAUTTAUUGGAGGAAUAUAUCUUCTT-3'.

### RNA extraction and qRT-PCR

Total RNA was isolated from tissue samples and cells using Trizol reagent (Invitrogen), and 2 μg of total RNA was reverse transcribed to obtain first-strand cDNA using an RNA-PCR kit (Takara) following the manufacturer's protocols. The resulting cDNA was used for real-time RT-PCR using a SYBR Green PCR Master Mix kit (Applied Biosystems) according to the manufacturer's instructions. The primers for EVI1 were: forward, 5′-GATTGCAGAACCCAAGTCAAGT-3′ and reverse, 5′-CTATTGGCGCCAAAATAGTCAG-3′. The primers for PTEN were: forward, 5′-GACCAGAGACAAAAAGGGAGTA-3′ and reverse, 5′-ACAAACTGAGGATTGCAAGTTC-3′. Relative quantification was performed by the 2-ΔΔCt method.

### Western blot analysis

Cell lysates were prepared by using RIPA lysis buffer (Beyotime, China), and the total protein concentration was determined by using a BCA protein detection kit (Pierce Biotechnology). Target proteins were separated by 8-15% sodium dodecyl sulfate-polyacrylamide gel electrophoresis (SDS-PAGE) and then transferred to polyvinylidene fluoride (PVDF) membranes. After blocking with 5% bovine serum albumin for 1 h, the PVDF membranes were incubated with primary antibodies at 4°C overnight. The following antibodies were used: anti-EVI1 (album, ab124934); anti-MECOM (ATLAS ANTIBODIES, HPA046537); anti-Lamin B (Beyotime, AF1408); anti-GAPDH (Beyotime, AF1186); anti-Akt (Cell Signaling Technology, 4691); anti-p-Akt (Cell Signaling Technology, 4060); anti-Cyclin A (Cell Signaling Technology, 4656); anti-p21 (Cell Signaling Technology, 2947); anti-CDK2 (Cell Signaling Technology, 2546); anti-Bcl-2 (Cell Signaling Technology, 4223); anti-Bax (Cell Signaling Technology, 5023); anti-Caspase 3 (Cell Signaling Technology, 9665); and anti-PTEN (Cell Signaling Technology, 9188). After washing with TBST, the membranes were incubated with secondary antibodies for 1 h. Signals were detected with an ECL detection reagent (Beyotime).

### Cell proliferation and colony formation

For a cell proliferation assay, transfected cells were seeded in 96-well plates at 3×10^3^ cells per well and incubated at 37°C for 24 h, 48 h, 72 h, 96 h or 120 h. Then, 10 μl of cell counting kit-8 (CCK-8) reagent (Dojindo, Kumamoto, Japan) was added to each well and incubated at 37°C for 1 h. The absorbance value at 450 nm was detected by a microplate reader. For a colony formation assay, 500 cells per well were plated in 6-well plates and incubated in DMEM supplemented with 10% FBS for 14 days. The colonies were fixed with methanol and stained with 0.5% crystal violet. Then, the visible colonies were counted.

### EdU pulse-chase incorporation

A 5-ethynyl-2'-deoxyuridine (EdU) assay was performed to assess cell proliferation by using the Cell-Light EdU DNA Cell Proliferation Kit (RiboBio, China). Transfected cells were seeded in 30-mm Petri dishes and cultivated to a normal growth stage. Then, cell fixation, permeabilization and EdU detection were performed following the manufacturer's instructions for a Click-iT™ EdU flow cytometry kit (Invitrogen). EdU-labelled cells were manually counted in ten randomly selected fields for each dish, and the percentages were calculated.

### Flow cytometric analysis of the cell cycle and apoptosis

For cell cycle analyses, transfected cells were collected and washed twice with phosphate-buffered saline. Then, the centrifuged cells were fixed in 75% ethanol at 4°C overnight. The cells were centrifuged again, stained using PI/RNase Staining Buffer (BD Bioscience Pharmingen, USA), and then analysed using flow cytometry. Cell apoptosis was detected using the PE Annexin V Apoptosis Detection Kit (BD Bioscience Pharmingen, USA). Cells contained in the supernatant were harvested and centrifuged. The cells were re-suspended in a binding buffer containing PE-Annexin and 7-AAD according to the manufacturer's instructions. The percentages of apoptotic cells were analysed by flow cytometry.

### TUNEL assays

A terminal deoxynucleotidyl transferase (TdT)-mediated dUTP nick-end labelling (TUNEL) assay was also used to assess cell apoptosis. Transfected cells were seeded in 30-mm petri dishes and cultivated until they filled the petri dishes. Cell fixation, permeabilization and TdT incubation were performed following the manufacturer's instructions for the TUNEL Apoptosis Detection Kit (Alexa Fluor 647) (Yeasen, Shanghai, China).

### In vivo tumourigenicity assays

QBC939 cells (4×10^6^) transfected with shEVI1 or a negative control (NC) were subcutaneously injected into the right flank of female BALB/c nude mice (6-8 weeks of age). Each group contained 6 mice. Tumour size was measured every 3 days. Tumour volume was determined using the following formula^16^: V = length×width^2^/2. The mice were sacrificed on the 21^st^ day after injection, and the xenograft tumours were removed and weighed. The Animal Welfare Committee of Shandong University approved all procedures involving animals.

### Immunohistochemical staining

A two-step approach was used for immunohistochemistry (IHC). After microwave antigen retrieval, anti-EVI1 or anti-PTEN primary antibodies were incubated with microarray and xenograft tissue sections overnight at 4℃. After washing, the tissue sections were incubated with biotinylated goat anti-rabbit antibodies at room temperature for 30 minutes. After DAB staining and haematoxylin counterstaining, the tissue sections were mounted in neutral gum and observed with a bright-field microscope. The staining intensity was evaluated as follows: strongly positive samples (scored as +++) had dark brown staining in more than 50% of tumour cells, and the staining completely covered the cytoplasm; moderately positive samples (scored as ++) had dark brown staining in 25-50% of tumour cells, whose cytoplasm was blurred; weakly positive samples (scored as +) had less brown staining of the cytoplasm; and "absence" (scored as -) defined samples with no significant staining in tumour cells. Strong and moderate scores were considered positive results. The TMA was scanned by HistoRx PM-2000™ and analysed by AQUAnalysis software.

### Statistics

Statistical tests were performed by using SPSS 17.0 statistical software packages (IBM, Armonk, NY, USA). Results are expressed as the mean±standard deviation (SD). All experiments were repeated three times, unless otherwise specified. The correlations between EVI1 and clinicopathological characteristics were analysed by the χ^2^ test. The correlation between EVI1 and PTEN was detected by linear correlation analysis. An independent-sample t test was used to compare differences between two groups. One-way ANOVA was used to analyse the differences among three groups. If there were significant differences, multiple comparisons were made between the groups. Univariate and multivariate Cox regression analyses were used to analyse survival data. P-values were considered statistically significant at less than 0.05.

## Results

### EVI1 expression is upregulated in HCCA tissue and related to a poor prognosis

**Figure 1:**
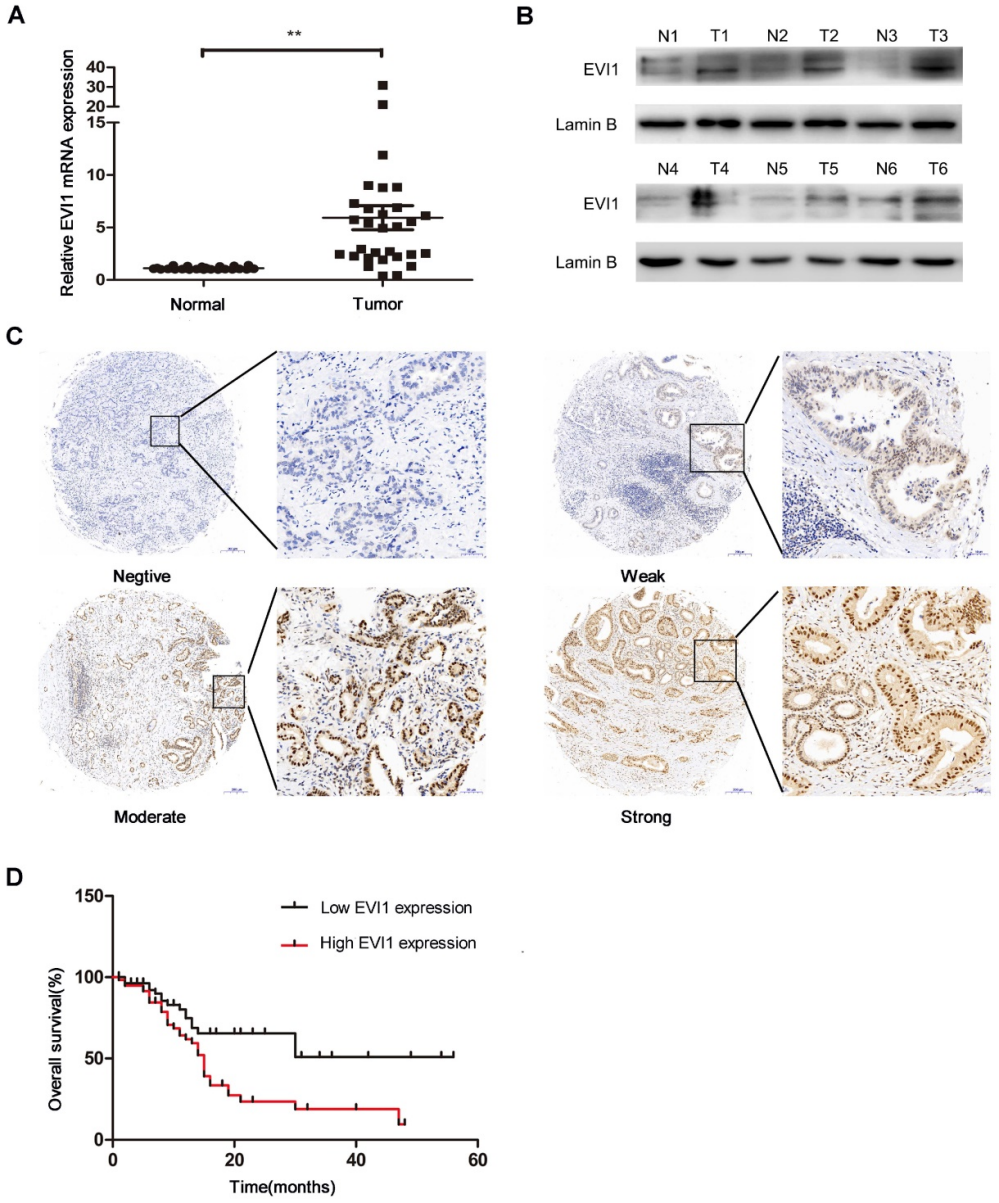
** Expression level of EVI1 in HCCA tissue samples and the relationship between EVI1 and overall survival.** (A) EVI1 expression in 30 pairs of HCCA tissue and adjacent normal bile duct tissue. (B) Protein expression of EVI1 in HCCA tissue samples detected by Western blotting. (C) Representative immunohistochemical staining for EVI1 in 114 HCC samples. (D) Kaplan-Meier survival analysis of patients with HCCA stratified by EVI1 expression. Overexpression of EVI1 was associated with decreased overall survival in the patients with HCCA. Data are presented as the mean±SEM and were analysed with a paired-sample t test. **P<0.01, N=normal, T=tumour.

To explore the role of EVI1 in HCCA, we detected the expression level of EVI1 in 30 pairs of HCCA tissue and adjacent normal bile duct tissue by quantitative real-time PCR (qRT-PCR) and Western blotting. We found that the mRNA level of EVI1 was significantly higher in the HCCA samples than in the noncancerous matched samples (P=0.0002, Figure 1A). Additionally, we found that EVI1 expression was also upregulated in the HCCA tissue samples compared with the corresponding adjacent normal tissue samples at the protein level (Figure 1B). EVI1 expression was further analysed in an HCCA TMA. We found that EVI1 was overexpressed in the HCCA tissue samples (Figure 1C) and was correlated with poor overall survival (OS) (P= 0.0042, Figure 1D). Together, these results revealed that EVI1 expression was significantly upregulated in HCCA and that overexpression of EVI1 was associated with a poor prognosis.

### Correlations between EVI1 and HCCA clinical parameters

We analysed the correlations between EVI1 and clinicopathological factors of HCCA. As shown in Table 1, high expression of EVI1 was markedly related to advanced tumour histological grade and increased tumour size (P=0.008 and P=0.023, respectively). EVI1 expression was not associated with age, sex, tumour depth, lymphatic metastasis, or TNM stage (all P> 0.05, Table 1). Then, we assessed the predictive value of EVI1 using the Cox‐proportional hazards model (Table 2). Univariate Cox regression analyses of OS demonstrated that histological grade (P=0.039), tumour size (P=0.004), lymphatic metastasis (P=0.011), TNM stage (P=0.001) and EVI1 expression (P=0.007) were significantly associated with the risk of death. Multivariate Cox regression analysis confirmed that EVI1 expression (P = 0.017) was an independent prognostic indicator for OS in HCCA, as was TNM stage (P = 0.013, Table 2).

### EVI1 promotes HCCA cell proliferation in vitro

**Figure 2:**
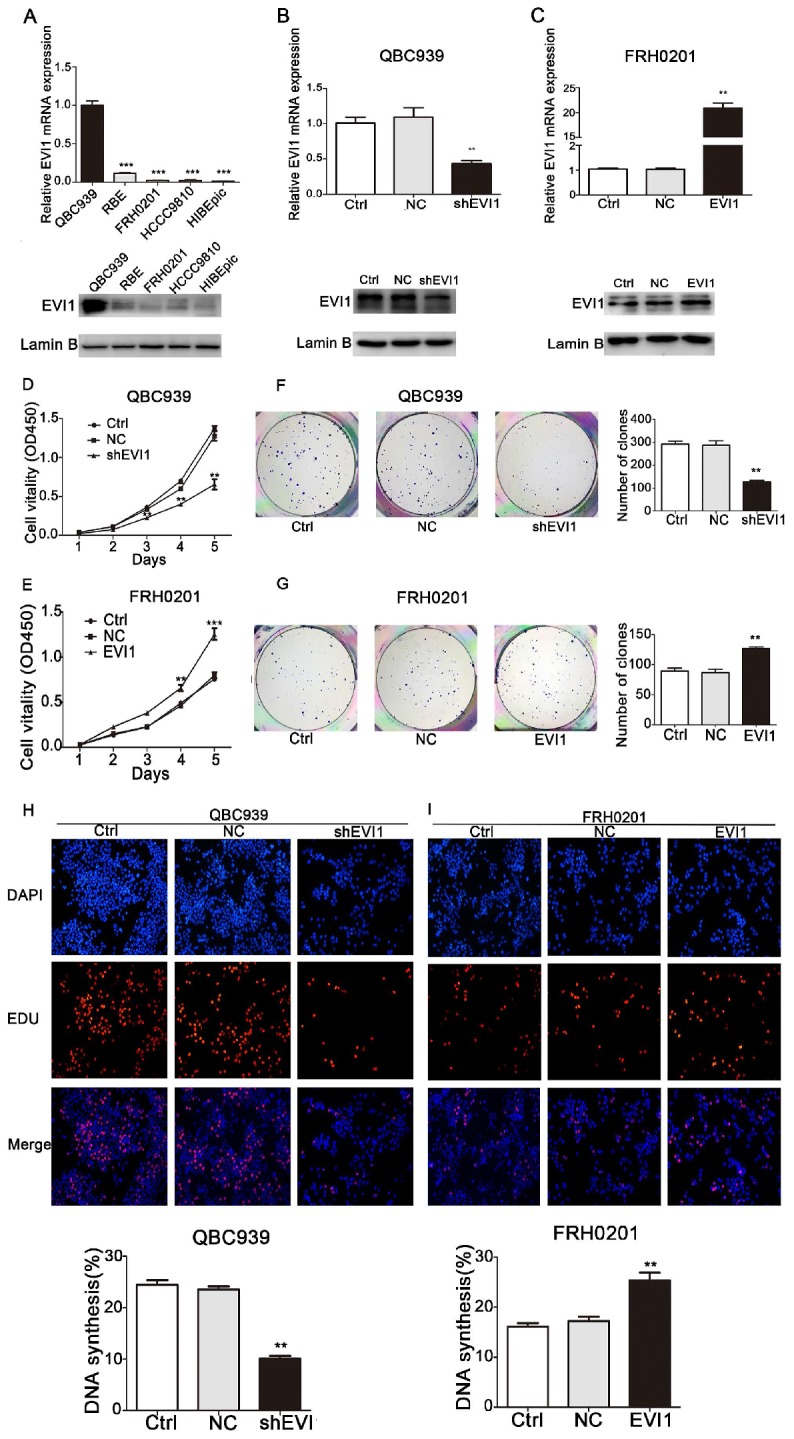
**EVI1 promotes the growth of HCCA cells.** (A) Quantitative real-time PCR (qRT-PCR) and Western blot analyses of EVI1 expression in CCA cell lines and human intrahepatic biliary epithelial cells (HIBEpiC). (B) Knocked down EVI1 expression in QBC939 cells detected by qRT-PCR and Western blotting. (C) FRH0201 cells transduced with an EVI1-overexpression plasmid. The overexpression efficiency was detected by qRT-PCR and Western blotting. (D, F and H) CCK-8 assays, colony formation assays and EdU assays to detect the proliferation of QBC939 cells following shRNA transfection. (E, G and I) FRH0201 cell proliferation analysed by CCK-8, colony formation and EdU assays following transfection with the EVI1-overexpression plasmid. Data are presented as the mean±SEM. **P<0.01, ***P<0.001, Ctrl=without transfection, NC=transfected with a noncoding shRNA, shEVI1= transfected with an EVI1-specific shRNA, and EVI1= transfected with the EVI1-over-expression plasmid.

To investigate the biological functions of EVI1 in HCCA, the expression of EVI1 was detected in 4 CCA cell lines and one HIBEpiC line by qRT-PCR and Western blotting. As shown in Figure 2A, the expression of EVI1 was highest in QBC939 cells and was significantly lower in FRH0201 cells. Then, QBC939 cells and FRH0201 cells were used for further functional studies. We constructed a stable EVI1-knockdown cell line (QBC939 cells) by transfection of lentiviral particles carrying an EVI1-specific shRNA. The mRNA level of EVI1 was remarkably decreased in the shEVI1 group compared with the control (Ctrl) and NC groups (P=0.0052, Figure 2B). This downregulation of EVI1 expression at the protein level was confirmed by Western blotting (Figure 2B). A CCK-8 assay showed that knocking down EVI1 expression resulted in significantly suppressed cell proliferation in the shEVI1 group compared with that in the Ctrl and NC groups, (Figure 2D). Similar results were observed in a colony formation assay and an EdU assay (Figure 2F and H). To further explore the function of EVI1 in tumour proliferation, we transfected FRH0201 cells with an EVI1-overexpression plasmid. The overexpression efficiency was detected by qRT-PCR and Western blotting (Figure 2C). The results showed that EVI1 overexpression promoted cell proliferation (Figure 2E, G and I). Therefore, these results demonstrate that EVI1 may play an important role in HCCA cell proliferation.

### Knocking down EVI1 expression blocks cell cycle progression and promotes apoptosis

**Figure 3:**
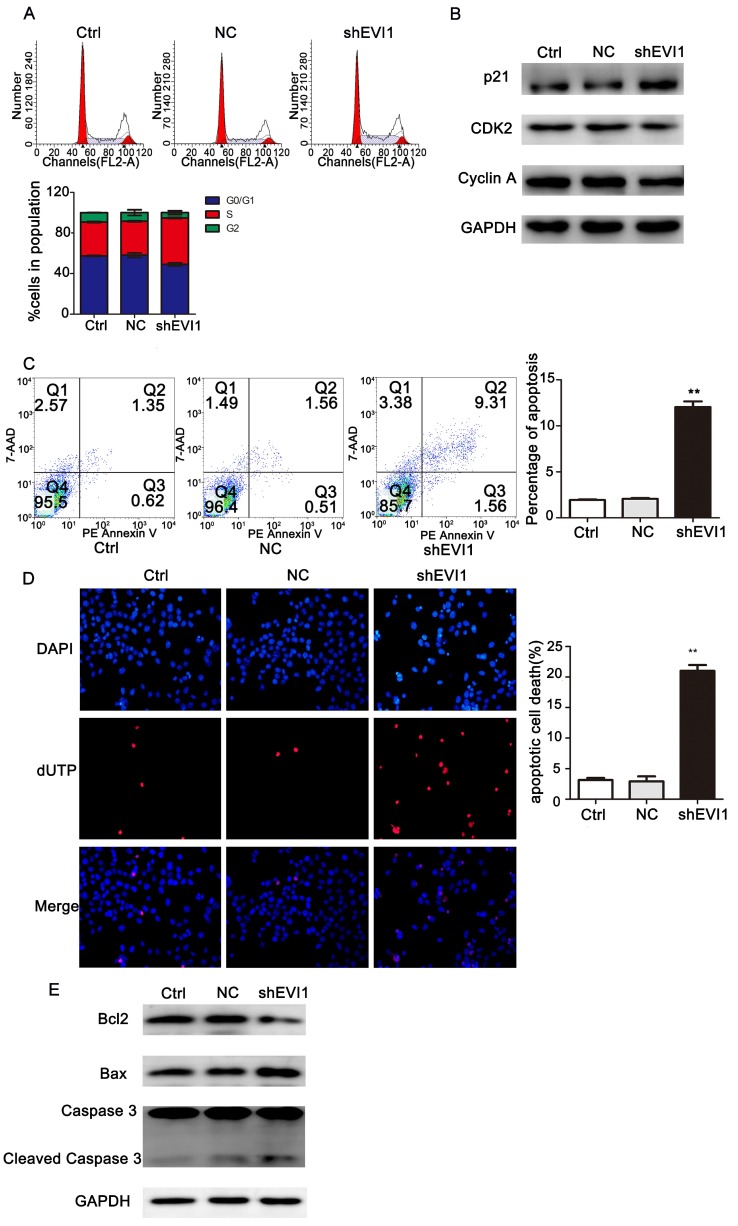
** Knocking down EVI1 expression affects cell cycle progression and apoptosis regulation in QBC939 cells. EVI1 suppresses the protein expression of PTEN and thus modulates the PI3K/AKT pathway.** (A) Flow cytometry analysis of the cell cycle was performed to determine the cell cycle distribution after transfection. (B) Western blot analysis of p21, CDK2 and Cyclin A expression in transfected QBC939 cells was performed. (C) Flow cytometry analysis of apoptosis was performed to evaluate cell apoptosis after transfection. (D) A TUNEL assay was carried out to assess apoptosis. (E) BCL2, Bax and Caspase 3 expression was measured by Western blotting. Data are presented as the mean±SEM. **P<0.001, Ctrl=without transfection, NC= transfected with a noncoding shRNA, shEVI1= transfected with an EVI1-specific shRNA.

To investigate the mechanism underlying the suppression of cell proliferation observed after silencing EVI1, we assessed the effects of knocking down EVI1 expression on the cell cycle and apoptosis by flow cytometry. As shown in Figure 3A, the percentage of S-phase cells was obviously higher in the shEVI1 group than in the Ctrl and NC groups (S%: 45.77% vs. 33.33% or 33.93%, respectively, P =0.000), implying that knocking down EVI1 expression might cause S-phase arrest in QBC939 cells. To further characterize the observed S-phase arrest, we examined the levels of several known S-phase cell cycle regulatory factors. Western blot analysis demonstrated that shEVI1 significantly increased p21 protein levels and decreased CDK2 and Cyclin A protein levels in QBC939 cells. The percentage of apoptotic cells in the shEVI1 group was notably increased, as determined by using Annexin V-PE staining for flow cytometry analysis (Figure 3C, shEVI1: 12.04%, Ctrl: 1.94%, and NC: 2.06%). TUNEL assays produced the same results (Figure 3D). Subsequently, we detected changes in apoptosis-related proteins by Western blotting. Knocking down EVI1 expression decreased the expression of Bcl-2 and increased the expression of Bax and cleaved caspase-3 (Figure 3E). Collectively, these results indicated that the downregulation of EVI1 expression markedly affected the cell cycle and boosted apoptosis.

### PTEN expression inversely correlates with EVI1 expression in HCCA

**Figure 4:**
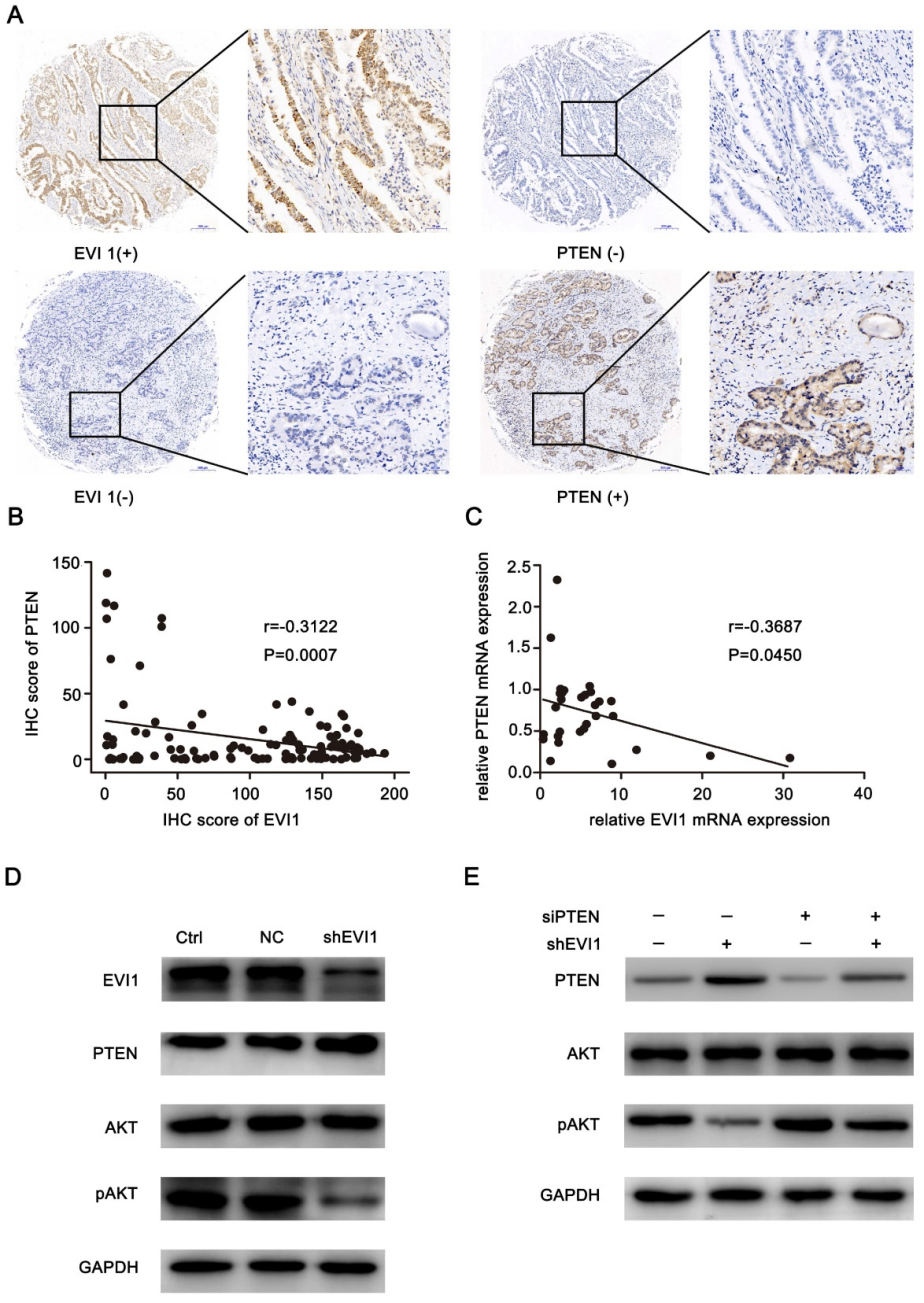
** PTEN expression is inversely correlated with EVI1 expression in human HCCA tissue.** (A) PTEN expression was low in EVI1-positive tissue samples and high in EVI 1-negative tissue samples. (B) The correlation between EVI1 and PTEN expression in TMA clinical samples was determined. (C) The correlation between relative EVI1 mRNA expression (the data in Figure 1A) and relative PTEN mRNA expression was determined in 30 pairs of HCCA tissue and adjacent normal bile duct tissue. (D) Comparisons of PTEN and AKT signalling pathways were analysed by Western blotting. (E) QBC939 cells transfected with NC or shEVI1 were treated with siPTEN. After 48 h of treatment, cell lysates were harvested for Western blotting. Western blot analysis showed that the repression of pAKT expression mediated by shEVI1 could be partially rescued by siPTEN. Ctrl=without transfection, NC= transfected with a noncoding shRNA, shEVI1= transfected with an EVI1-specific shRNA, siPTEN= transfected with a PTEN-specific siRNA.

To analyse the mechanism by which EVI1 promotes the progression of HCCA, we evaluated EVI1 and PTEN expression in an HCCA TMA. We found that low expression of PTEN mainly occurred in the tumour tissue samples with high EVI1 expression, and high expression of PTEN mainly occurred in the tumour tissue samples with low EVI1 expression (Figure 4A). A statistically significant inverse correlation was observed between the EVI1 and PTEN proteins (r=-0.3122, P=0.0007, Figure 4B). This result was also verified by qRT-PCR analysis of fresh tumour tissue samples (r=-0.3687, P=0.045, Figure 4C).

### Knocking down EVI1 expression promotes the expression of PTEN and represses the phosphorylation of AKT

**Figure 5:**
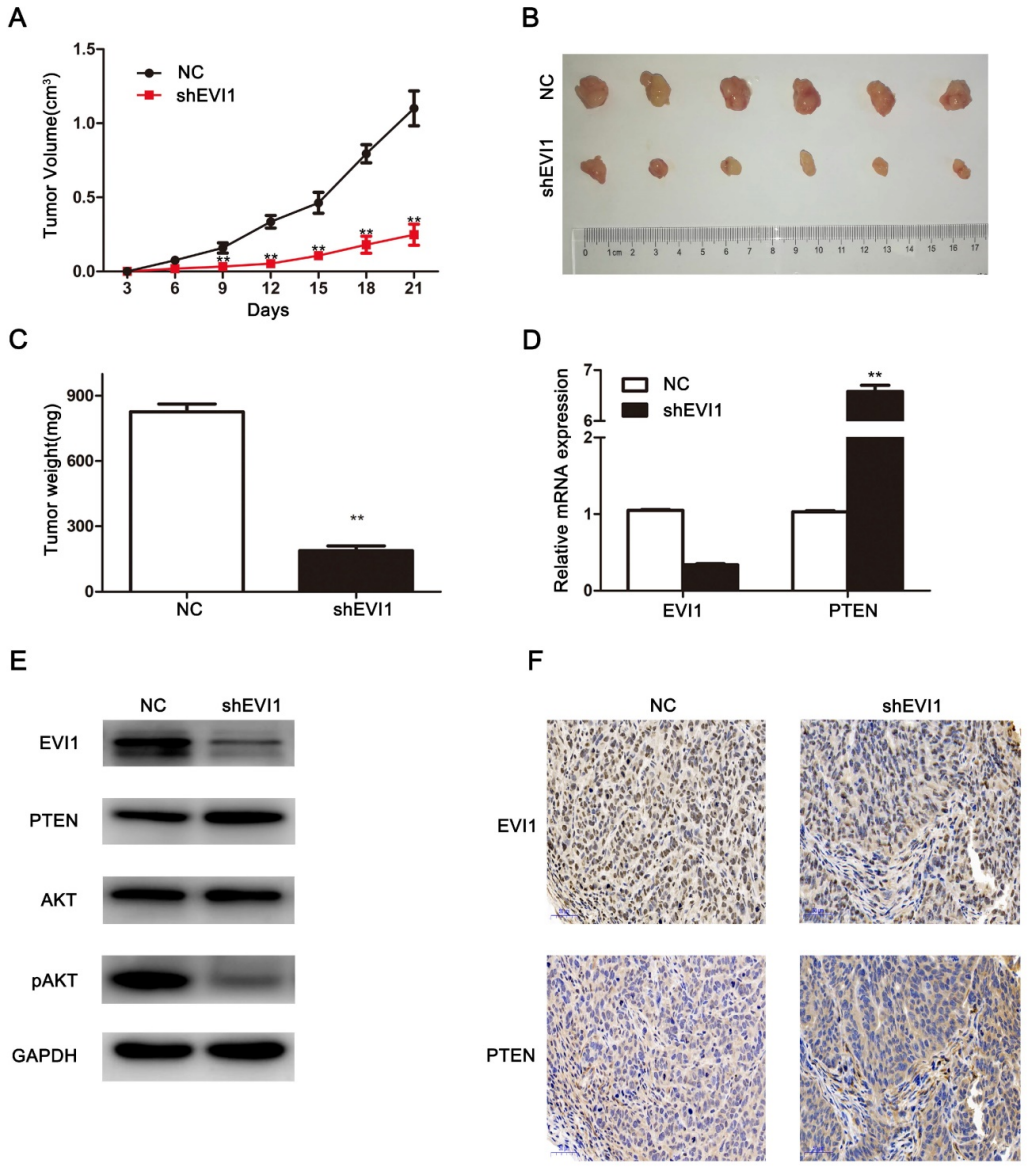
** Knocking down EVI1 expression reduces the growth of HCCA tumour xenografts.** (A) The tumourigenicity of EVI1 knockdown cells and their counterparts in nude mice was determined. Nude mice were subcutaneously transplanted with cells transfected with NC or shEVI1. Beginning on the third day, tumour volumes were measured every 3 days, and tumour growth curves were plotted until the mice were euthanized on day 21. (B) All animals were sacrificed on the 21st day after tumour cell injection, and the tumours were photographed. (C) Tumours were weighed when the mice were sacrificed. (D) QRT-PCR was used to analyse EVI1 and PTEN expression in the xenografts. (E) EVI1, PTEN, AKT and pAKT expression in the nude mouse tumours was measured by Western blotting. (F) The in vivo relationship between EVI1 and PTEN was shown by immunohistochemical staining of the transplanted tumours from nude mice. Data are presented as the mean±SEM and were analysed with an independent-sample t test. **P<0.01, NC= transfected with a noncoding shRNA, shEVI1= transfected with an EVI1-specific shRNA.

We assessed the effect of knocking down EVI1 expression on PTEN protein expression in vivo and in vitro. As shown in Figure 4D and Figure 5E, the expression of PTEN was significantly increased by knocking down EVI1 expression. Since AKT is an important target of PTEN and PTEN negatively regulates the phosphorylation of AKT^17^, the expression of phosphorylated AKT was also examined. Western blot analysis indicated that AKT phosphorylation was significantly reduced by knocking down EVI1 expression in vivo and in vitro (Figure 4D and Figure 5E). In PTEN-depleted cells, the inhibitory effect of EVI1 on pAKT was attenuated (Figure 4E). These results indicated that EVI1 could regulate the AKT signalling pathway by regulating PTEN in HCCA. These results suggest that downregulation of PTEN expression by EVI1 may contribute to EVI1-induced tumour carcinogenesis and development.

### Knocking down EVI1 expression significantly inhibits tumour growth in vivo

To further investigate the biological effects of EVI1 on HCCA tumourigenesis in vivo, we subcutaneously injected cells (shEVI1- or NC-transfected cells) into nude mice and monitored tumour growth. As shown in Figure 5A, compared with NC transfection, shEVI1 transfection significantly inhibited tumour growth. In addition, the average tumour weight was remarkably lower in the shEVI1 group than in the NC group (P<0.01, Figure 5C). Furthermore, the expression of PTEN in the shEVI1 group was higher than that in the NC group, as determined by qRT-PCR and Western blotting (Figure 5D and E). The expression of pAKT was also decreased in the shEVI1 group compared with the NC group (Figure 5E). These results were consistent with the results obtained in vitro. These results further confirm that downregulation of EVI1 expression suppresses the development of HCCA.

## Discussion

Due to the latent genesis and development of HCCA, it is difficult to diagnose and treat this disease in the early phase. There are no effective treatments for advanced HCCA. Therefore, the prognosis of advanced-stage patients is very poor^6^, ^18^, ^19^. EVI1 has been confirmed to participate in solid tumour genesis and development^20^. When inappropriately expressed, EVI1 is considered an independent poor prognostic factor^21^. In particular, a recent study found that EVI1 was overexpressed and associated with a poor prognosis in ICC^13^. However, the status of EVI1 expression in HCCA is still unknown.

In the present research, we focused on the expression levels of EVI1 and its biological function in HCCA. Our study showed that EVI1 was overexpressed in HCCA tissue compared with adjacent normal bile duct tissue. Furthermore, we investigated the relationships between EVI1 expression and clinicopathological characteristics in HCCA. We found that high EVI1 expression was correlated with a low histological grade and large tumours and predicted poor OS. Multivariate analysis showed that EVI1 was an independent prognostic factor in HCCA patients. In acute myeloid leukaemia, EVI1 overexpression is an independent adverse prognostic factor^22^, ^23^. Similar results have also been reported in ICC^13^, hepatocellular carcinoma^24^, colorectal cancer^25^, prostate cancer^26^, lung cancer^27^, glioblastoma multiforme^28^ and so on^29^, ^30^. These data indicate that EVI1 plays an important role in tumour progression.

Queisser et al. reported that downregulation of EVI1 expression suppressed cell growth and induced cell cycle arrest and apoptosis in prostate cancer^26^. Wang et al. found that EVI1 silencing reduced cell proliferation and apoptosis resistance in breast carcinoma^31^. In pancreatic cancer cells, EVI1 depletion remarkably inhibits cell growth and migration, indicating its oncogenic roles^32^. To explore the biological function of EVI1 in HCCA cells, we constructed a stable EVI1-knockdown cell line with lentiviral particles. We demonstrated that knocking down EVI1 expression inhibited HCCA cell proliferation both in vitro and in vivo. Furthermore, overexpression of EVI1 could promote tumour cell growth in vitro. Downregulated EVI1 expression blocked the cell cycle in S phase and promoted cell apoptosis. All of these results were consistent with those of the aforementioned studies.

Cell proliferation and apoptosis are regulated by multiple signalling pathways. The effects of the PI3K/AKT signalling pathway on proliferation and apoptosis have been proven in many kinds of cancers, including HCCA^33^-^36^. PTEN negatively regulates the PI3K/Akt pathway in cancer, exerting tumour suppressor activity^17^, ^37^, ^38^. PTEN has been reported to be a direct target of EVI1 and related to EVI1-mediated biological effects on cell proliferation and survival in human leukaemia cells^14^. In this study, we found a significant negative correlation between EVI1 and PTEN expression in tumour tissue samples. Therefore, we speculated that PTEN was an important target of EVI1 in HCCA. Further study showed that knocking down EVI1 expression increased the protein level of PTEN and reduced the phosphorylation of AKT in QBC939 cells. Moreover, downregulation of PTEN expression could attenuate the influence of EVI1 on the AKT pathway. These results indicated that EVI1, as a transcription factor, performed its oncogenic roles at least partially by repressing PTEN expression and activating AKT signalling pathways.

In conclusion, we demonstrated that EVI1 was overexpressed in HCCA tissue samples and cell lines and that its expression was negatively correlated with HCCA patient prognosis. In addition, our study suggested that EVI1 might regulate tumour cell proliferation by repressing PTEN and activating the AKT pathway. Therefore, EVI1 may be a potential target in HCCA treatment.

## Figures and Tables

**Table 1:** Correlations between EVI1 expression and clinicopathological characteristics of HCCA patients

Characteristic		Case(n)	EVI1 expression		χ*2* value		P-value	
			High (n)	Low (n)				
Age (years)								
	≤60	37	18	19	0.109		0.741	
	>60	77	40	37				
Gender								
	Male	81	38	43	1.759		0.185	
	Female	33	20	13				
Histological grade								
	Well	30	9	21	7.100		**0.008**	
	Moderately/ Poorly	84	49	35				
Tumor size (cm)								
	>2.5	53	33	20	5.139		**0.023**	
	≤2.5	61	25	36				
Tumor depth								
	T1	40	21	19		0.065		0.799
	T2- T4	74	37	37				
Lymphatic metastasis								
	Absent	75	39	36		0.111	0.739	
	Present	39	19	20				
TNM stage								
	I/II	73	40	33		1.246		0.264
	III/IV	41	18	23				
								

**Table 2:** Univariate and multivariate analyses of prognostic factors for overall survival in HCCA patients

Variables	Univariate analysis			Multivariate analysis		
	HR	95%CI	P value	HR	95%CI	P value
Age(>60 vs ≤60)	0.576	0.314-1.058	0.075			
Gender(Male vs Female)	1.108	0.624-1.969	0.726			
Histological grade (Well vs Moderately/ Poorly)	2.214	1.040-4.712	**0.039**	0.720	0.328-1.580	0.413
Tumor size(>2.5 vs ≤2.5)	0.442	0.252-0.776	**0.004**	1.749	0.980-3.119	0.058
Tumor depth(T1 vs T2-4)	1.289	0.694-2.394	0.421			
Lymphatic metastasis (Absent vs Present)	2.035	1.180-3.511	**0.011**	1.681	0.567-4.986	0.349
TNM stage(I/II vs III/IV)	2.437	1.412-4.206	**0.001**	0.254	0.083-0.780	**0.017**
EVI1 expression (High vs Low)	2.269	1.255-4.102	**0.007**	0.451	0.240-0.846	**0.013**

HR, hazard ratio; 95% CI, 95% confidence interval.
